# The Effect of “Novelty Input” and “Novelty Output” on Boredom During Home Quarantine in the COVID-19 Pandemic: The Moderating Effects of Trait Creativity

**DOI:** 10.3389/fpsyg.2020.601548

**Published:** 2020-12-14

**Authors:** Zheng Liang, Qingbai Zhao, Zhijin Zhou, Quanlei Yu, Songqing Li, Shi Chen

**Affiliations:** ^1^Key Laboratory of Adolescent Cyberpsychology and Behavior (Ministry of Education), School of Psychology, Central China Normal University, Wuhan, China; ^2^Key Laboratory of Human Development and Mental Health of Hubei Province, School of Psychology, Central China Normal University, Wuhan, China

**Keywords:** state boredom, trait creativity, novelty-seeking behavior, COVID-19, home quarantine

## Abstract

Governments have adopted strict home quarantine measures during the COVID-19 pandemic. A monotonous, barren, and under-stimulating environment can cause state boredom, and people often deal with boredom via novelty-seeking behavior. Novelty-seeking behavior can be divided into “novelty input” and “novelty output.” The former refers to obtaining novel information such as browsing the Web; the latter refers to engaging in creative behavior such as literary creation. This study explores the relationship between two types of novelty-seeking behavior and individual state boredom during home quarantine, along with the moderation effect of trait creativity. The study sample consists of 582 Chinese college students who were quarantined at home during the COVID-19 pandemic. Participants completed the Multidimensional State Boredom Scale, the Williams Creativity Aptitude Test, and self-compiled questionnaires of novelty input and novelty output. The results show that there is no significant relationship between novelty input or novelty output and boredom during the COVID-19 quarantine. Trait creativity is found to negatively moderate the relationship between the two means of novelty seeking and boredom. Specifically, novelty output negatively predicts the state boredom of individuals with high creativity, while novelty input positively predicts the state boredom of individuals with low creativity. Our findings suggest that different novelty-seeking behaviors may have different effects on the boredom level of individuals with high versus low creativity during quarantine. During a quarantine period, individuals should avoid excessively engaging in novelty input behaviors aimed at escaping boring situations.

## Introduction

Since December 2019, COVID-19 has spread to many countries and territories around the world. To limit the spread of the virus, governments have adopted strict containment measures, including lockdowns, “stay-at-home” orders, closed-off community management, etc. (Brooks et al., 2020; Gostin and Wiley, 2020; Pellegrini et al., 2020). Affected by these policies, the daily lives of billions of people have been changed (Lades et al., 2020; Matias et al., 2020). Except for buying necessary food or going to a doctor, people can only stay at home; most work and study activities have been suspended or converted to online forms (Pellegrini et al., 2020; Zhou, 2020). In many countries, as of summer 2020, the home quarantine policy is expected to continue for 6 months or 1 year until a vaccine is available (Matias et al., 2020).

Quarantine has been shown to be effective in slowing the spread of COVID-19 (Prem et al., 2020), as it can prevent people from interacting with individuals infected with the COVID-19 virus, thereby reducing the risk of illness. However, prolonged quarantine may give rise to psychological and emotional problems (Pellegrini et al., 2020; Qiu et al., 2020; Viana and de Lira, 2020). Numerous surveys have shown that people in quarantine environments report a strong sense of boredom (Cava et al., 2005; Reynolds et al., 2008; Brooks et al., 2020; Droit-Volet et al., 2020). As a negative compound emotion, boredom experience is aversive (Bench and Lench, 2013) and may impair individual attention and cognitive functions (Wallace et al., 2003; Vodanovich and Watt, 2016; Burn, 2017). In addition, the generation of boredom is often accompanied by the appearance of various negative emotions, such as depression and anxiety, that severely affect people’s mental state (Zhou et al., 2012). In terms of pandemic prevention and control, individuals who are high in boredom have been shown to have lower compliance with the quarantine policy and a higher likelihood of contracting COVID-19, which has been verified in samples from North America and Europe (Boylan et al., 2020; Wolff et al., 2020). Therefore, it is vital to explore strategies for coping with boredom during the pandemic quarantine period. Similar to anxiety and other emotions, boredom can be divided into two types – state boredom (an emotion that appears in a specific situation) and trait boredom (an individual’s propensity to experience feelings of disinterest) – where the former is more suitable for exploring strategies for coping with boredom (Vogel-Walcutt et al., 2012). Thus, the boredom explored in this article is specifically state boredom.

To date, researchers have explored boredom from the perspectives of cognition, arousal, and functionality (Todman, 2003; van Tilburg and Igou, 2011; Vogel-Walcutt et al., 2012). Cognitive theories of boredom suggest that when individuals experience it means that their cognitive resources are not optimally utilized during the current task (Danckert et al., 2018b). Boredom may be caused by monotonous situations in which cognitive resources are idle or difficult situations in which cognitive resources are insufficient (Skowronski, 2012; Westgate and Wilson, 2018). Arousal theories of boredom propose that boredom is the outcome of a mismatch between the arousal demand and the level of arousal provided by the environment (Raffaelli et al., 2018), including the experience of both high and low arousal (Merrifield and Danckert, 2014). Individuals’ self-reports of arousal states during boredom, and the high correlation between state boredom and sleepiness, support the view that boredom is a low-arousal experience (Game, 2007; Danckert et al., 2018a), which may be due to inadequate stimulation. However, when we try to change but can’t get rid of this monotonous circumstance, the boredom experience shows a high arousal state of restlessness (Eastwood et al., 2012; Danckert et al., 2018a). Different from cognitive theories and arousal theories that focus on external factors, functional theories of boredom propose that the individual’s judgment of the meaning of current activities underlies their sense of boredom (van Tilburg and Igou, 2011). Boredom can often arise from monotonous tasks that lack meaning (Barbalet, 1999), and boredom as a signal can prompt people to change their behavior or cognition (Elpidorou, 2014). For example, a study found that when participants of a monotonous task were told that their small reward for completing the experiment would be used to provide clean water for the poor, they reported less boredom compared to those who received the reward directly (Westgate and Wilson, 2018). Eastwood et al. (2012) combined the above theories to propose that boredom is a negative experience of desiring, but not being able to engage in, satisfactory activities.

In the context of home quarantine, many factors can induce boredom. First, the monotonous and constrained isolation environment leads to a lower degree of individual arousal, which may lead to boredom (Game, 2007; Burn, 2017; Danckert et al., 2018b). Second, due to the suspension of classes and work, most people do not engage in complex cognitive activities at home, and cognitive resources are not optimally used. Third, unstructured time at home can make people feel bored. Martin et al. (2006), through a qualitative study, found that when people retire at home or return home after a day’s work, a large amount of free time makes them experience a strong sense of boredom and the feeling of being “trapped in a cage.” According to the control-value theory of Pekrun et al. (2010), the low sense of control that an individual feels in unstructured time, as well as the sense of meaninglessness and valuelessness brought about by doing nothing may lead to boredom.

However, these situational factors may be only indirect factors of boredom. They greatly increase individuals’ chances of experiencing boredom, but they are not enough to directly cause boredom (Danckert et al., 2018b). Boredom will arise or increase when a person desires to engage in satisfactory activities but fails to do so, or engages in inappropriate behaviors. Conversely, when people adopt appropriate boredom coping strategies to respond to upcoming boredom signals, they may prevent boredom (Game, 2007; Nett et al., 2011; Haager et al., 2016). If the boredom during the quarantine is not well alleviated then the result seems to be serious. Compliance with the quarantine policy is positively correlated with self-control (Wolff et al., 2020), individuals with low self-control are more likely to be bored (Struk et al., 2016; Isacescu et al., 2017; Danckert et al., 2018a; Isacescu and Danckert, 2018; Mugon et al., 2018), which makes them more likely to break the quarantine rules (Boylan et al., 2020). Therefore, it is meaningful to explore appropriate boredom coping strategies during the quarantine.

Novelty seeking refers to a tendency to explore new and unfamiliar things (Costa et al., 2014). Previous studies have found that individuals adopt novelty-seeking behavior as a way to cope with boredom (Schweizer, 2006; Danckert et al., 2018b; Sharp et al., 2018). On the one hand, individuals improve their arousal levels by acquiring some novel stimuli, thereby alleviating boredom (Mercer-Lynn et al., 2014; Burn, 2017; Yang et al., 2020). For example, employees often use the Internet to obtain novel information to deal with workplace boredom (Pindek et al., 2018), students may use their mobile phone to obtain novel information to deal with academic boredom (Eren and Coskun, 2016; Yang et al., 2020), and at home, watching TV is a common way for people to cope with family boredom (Martin et al., 2006). Bench and Lench (2019) found that, compared to continuing to view neutral pictures, individuals are more likely to choose novel pictures (even negative ones) to relieve boredom.

On the other hand, individuals may change their boring environment or enhance their interest in current activities by engaging in creative behaviors (Skowronski, 2012). If the current task or situation can be changed, the boredom experienced by the individual can be reversed immediately (Sharp et al., 2018). Schubert (1977) found that when individuals complete problem-solving tasks, as the duration of the task increases they react more creatively to avoid boredom. Sansone et al. (1999) found that participants make certain tasks more interesting by changing their handwriting creatively and artistically. Game (2007) found that in order to cope with workplace boredom or to enrich their free time, those who cope well with boredom adopt more creative behaviors to complete tasks. Some studies have found that individuals who have experienced boring activities perform better in subsequent creativity tasks (e.g., Gasper and Middlewood, 2014; Mann and Cadman, 2014). State boredom seems to bolster creativity; however, trait boredom cannot predict individual creativity (Hunter et al., 2016). Considering the impact of boredom on individuals’ cognitive function, it seems strange to think that people are more creative when they are bored (Danckert et al., 2018b). Haager et al. (2016) suggested that when individuals carried out creative tasks the boredom-inducing environments in the experiment were no longer boring. Therefore, it seems reasonable to regard creative behavior as a way for individuals to avoid boredom.

In short, novelty-seeking behaviors for the purpose of coping with boredom can be roughly divided into novel stimulus input (referred to here as “novelty input”) and novel behavior output (referred to here as “novelty output”). Novelty input refers to alleviating boredom through the acquisition of novel information, wherein the individual shifts attention to a novel, exciting, and high-arousal situation. This behavior can overcome the discomfort caused by boredom (Sharp et al., 2018), but does not substantially change the boring environment, and thus comprising a kind of behavioral-avoidance strategy (Nett et al., 2010; Zhou and Kam, 2017). Novelty output refers to alleviating boredom by creating novel products or completing a task in an innovative way. This behavior directly and constructively manages the source of boredom (Game, 2007) and changes the actual boredom situation, thus comprising a kind of behavioral-approach strategy (Nett et al., 2010; Zhou and Kam, 2017). During quarantine, individuals may cope with boredom through both novelty input (e.g., browsing the Web) and novelty output (e.g., literary creation).

However, adopting behavioral strategies to alleviate boredom is not always effective. Eren (2016) found that the adoption of behavioral-approach strategies is not significantly negatively related to the boredom of prospective teachers in the classroom environment. Nett et al. (2011) even found that behavioral-approach strategies and behavioral-avoidance strategies lead to an increase in students’ academic boredom, and that individuals appear to have different preferences for the two kinds of boredom coping strategies. These results indicate that the same boredom coping strategy may have different effects on different individuals; this may be caused by differences in personal traits (Game, 2007). We speculate that individual trait creativity may moderate the relationship between novelty-seeking behavior and boredom during quarantine.

Because individuals with high creativity have the tendency to explore and the characteristic of acting appropriately in unstructured situations (Gostoli et al., 2017), they are more likely to gain a sense of meaning in creative behavior compared to individuals with low creativity. In this process, highly creative individuals also have better arousal and utilization of cognitive resources. Therefore, compared with novelty input, novelty output may be more effective to relieve boredom in this group.

Although individuals with low creativity have a strong desire for novelty, they lack the ability to transform this desire into creative behavior or products. Novelty output may thus consume their significant cognitive resources. In addition, since their desire to explore and try is low (Gostoli et al., 2017), novelty input in its main form of obtaining novel external stimuli may be a better way to improve their arousal. Therefore, compared with novelty output, novelty input may be more effective to relieve boredom in these individuals.

The current study aims to investigate whether novelty input and novelty output are associated with the state boredom of individuals during home quarantine, and whether such associations are moderated by trait creativity. Based on these aims, we propose the following hypotheses:

***Hypothesis 1***. Both novelty input and novelty output are negatively correlated with state boredom during home quarantine.
***Hypothesis 2***. Trait creativity moderates the relation between novelty input and state boredom during home quarantine.
***Hypothesis 3***. Trait creativity moderates the relation between novelty output and state boredom during home quarantine.

## Materials and Methods

### Participants and Procedure

This study investigates the home quarantine situation of participants during early stages of the COVID-19 pandemic, in February and March 2020. The data were collected via an online survey during April and May 2020. Participants were required to indicate their demographic information, the average number of times they left home per week during quarantine, and the strictness of local closed-off community management; they then completed four questionnaires. The research was approved by the Ethics Institutional Review Board of Central China Normal University.

Participants included 644 students from China who were selected using convenience sampling. A total of 62 participants were excluded prior to data analysis because their answer times were too short (less than 200 s) or because they indicated that they were not under home quarantine before. The final analytic sample consisted of 582 college students and graduate students from 26 provinces in China (178 male; age range 18–28; *M*_age_ = 20.82, *SD*_age_ = 2.13), with an effective recovery rate of 90.37%.

### Measures

#### State Boredom

The Multidimensional State Boredom Scale (MSBS) is a self-reported state boredom measurement tool developed by Fahlman et al. (2013) for college students in general situations. We used the Chinese version of the MSBS (Liu et al., 2013) to measure the state boredom of participants during the home quarantine period. The scale has 24 items and uses a 7-point scale (completely disagree–completely agree). Responses to all 24 items were summed to obtain the MSBS score, with higher scores indicating higher levels of state boredom. In previous studies, the scale has shown good reliability and validity (Liu et al., 2013; Ng et al., 2015; Zhao et al., 2016). In the present study, the Cronbach’s alpha was 0.956.

#### Trait Creativity

Trait creativity was measured using the Williams Creativity Aptitude Test (WCAT), which is part of the Creativity Assessment Packet (Williams, 1980). We used the Chinese version developed by Lin and Wang (1997), which consists of 50 items and four dimensions: curiosity (inclination to explore, or play with an idea), imagination (predisposition to visualize and construct mental images, or feel intuitively), challenge (the tendency to look for new alternatives and solutions to problems, to restore order out of chaos), and risk-taking (the inclination to act under unstructured conditions and to defend one’s own ideas) (Gostoli et al., 2017; Sica et al., 2017). All items range from 1 (completely disagree) to 3 (completely agree), except for eight items with an inverse direction. Responses to all 50 items (reverse-item scores were subtracted from four prior to calculation) were summed to obtain the WACT score, with higher scores indicating a greater aptitude for creativity (He et al., 2018). The four subscale scores were calculated by summing responses to items for each subscale (Tong et al., 2015). In previous studies, the scale has shown good reliability and validity (Jin et al., 2016; Sica et al., 2017; He et al., 2018). In the present study, the Cronbach’s alpha of each subscale in this study ranged from 0.638 to 0.794.

#### Novelty Input

As noted above, in this study novelty input refers to situations in which individuals obtain novel information through various channels during home quarantine. Considering that people are frequently using the Internet to obtain information during the quarantine period (Eidi and Delam, 2020), we compiled a novelty input questionnaire based on “The 45th China Statistical Report on Internet Development” (China Internet Network Information Center, 2020) and our prior interviews. The questionnaire is divided into three dimensions: film and television shows (e.g., watching movies, television series, documentaries, and other film and television works that have not been watched before), online platforms (e.g., browsing Chinese Quora, Sina Microblog, and other platforms to obtain novel information), and literary and artistic works (e.g., reading literary works that have not been read before). The questionnaire includes 10 items and uses a 5-point Likert scale ranging from 1 (never) to 5 (always). Responses to all 10 items were summed to obtain the novelty input score, with higher scores indicating more novelty input behaviors enacted on the part of the individual. The Cronbach’s alpha for the whole questionnaire was 0.744 and ranged between 0.603 and 0.654 for each dimension. The results of a confirmatory factor analysis revealed that the questionnaire had good construct validity (χ^2^/df = 2.407, CFI = 0.954, TLI = 0.936, RMSEA = 0.049, SRMR = 0.040). These findings demonstrate that the novelty input questionnaire is a reliable and valid tool.

#### Novelty Output

Again, as noted above, novelty output in this study refers to situations in which individuals engage in daily creative behaviors to produce novel ideas or products during home quarantine. We compiled our novelty output questionnaire based on our prior interviews and the Biographical Inventory of Creative Behaviors (BICB) (Batey, 2007). The questionnaire is divided into five dimensions: domestic creativity (e.g., redesigned and redecorated a bedroom, kitchen, personal space, etc.), artistic creativity (e.g., written a short story), scientific creativity (e.g., proposed a theory to explain a phenomenon), leadership creativity (e.g., mentored/coached someone else to improve their performance), and entertaining creativity (e.g., invented a form of novel family entertainment). The items in the first four dimensions were selected from the representative items of each dimension of the BICB, and some items were reworded to fit and refer to the context of home quarantine. The items of the entertaining creativity dimension were compiled based on the contents of the prior interviews. The questionnaire includes 21 items and uses a 5-point Likert scale ranging from 1 (never) to 5 (always). Responses to all 21 items were summed to obtain the novelty output score, with higher scores indicating more novelty output behaviors enacted on the part of the individuals. The Cronbach’s alpha for the whole questionnaire was 0.910 and ranged between 0.723 and 0.825 for each dimension. The results of a confirmatory factor analysis revealed that the questionnaire had good construct validity (χ^2^/df = 3.290, CFI = 0.922, TLI = 0.908, RMSEA = 0.063, SRMR = 0.060). These findings demonstrate that the novelty output questionnaire is a reliable and valid tool.

### Data Analysis

The data for this study were processed using SPSS 24.0. First, we used Harman’s single-factor test (Podsakoff et al., 2003) to analyze whether there was any common method bias in our data. Second, scores from the four questionnaires were analyzed using descriptive statistics and Pearson bivariate correlation. Finally, the SPSS macro PROCESS introduced by Hayes (2013) was used to test the moderation model; the average times respondents left home per week during quarantine and the strictness of local closed-off community management were entered as covariates into the moderation model. For the significant effects, we employed a conventional method (pick-a-point approach) for plotting simple slopes to understand moderation effects, at one standard deviation below and above the mean.

## Results

### Common Method Biases

First, common variance analysis was applied to the four questionnaires through factor analysis. The chi-square of Bartlett’s test of sphericity reached significance. Following principal component analysis, 24 eigenvalues greater than 1 were extracted. The first factor to explain the variance was 12.541%, which is less than the 40% required by the critical standard (Podsakoff et al., 2003). These results suggest that common method bias is not a major concern in this study.

### Descriptive and Bivariate Correlations Analysis

Descriptive statistics and a correlation matrix of novelty input, novelty output, state boredom, and trait creativity are provided in Table 1. Results of bivariate correlation analysis show that novelty input, novelty output, and trait creativity were significantly and positively correlated with each other (*p* < 0.001). The correlation between novelty input or novelty output and state boredom was not significant (*p* > 0.05).

**TABLE 1:** Descriptive statistics and results of correlational analysis.

Variables	*N*	Mean	SD	1	2	3	4
1 Novelty input	582	32.75	6.12	1			
2 Novelty output	582	41.27	13.27	0.474**	1		
3 State boredom	582	89.22	30.44	0.020	−0.049	1	
4 Trait creativity	582	105.95	11.87	0.348**	0.344**	0.066	1

**p < 0.001.

### Moderation Effect of Trait Creativity on the Relationship Between Novelty Input and Boredom

The results of the moderation analysis with selected state boredom as the dependent variable, novelty input as an independent variable, and trait creativity (and its components) as a moderator are presented in Table 2.

**TABLE 2:** Results of moderation analysis with boredom as the dependent variable, novelty input as the independent variable, and trait creativity as the moderator.

Interaction effect	Coefficient	*SE*	*t*	*P*
**Trait creativity as moderating variable**				
NI × TC	−0.113	0.039	−2.870	0.004
**Indicators of trait creativity as moderating variables**				
NI × curiosity	−0.117	0.040	−2.934	0.004
NI × imagination	−0.119	0.038	−3.115	0.002
NI × challenge	−0.069	0.042	−1.650	0.100
NI × risk-taking	−0.066	0.040	−1.678	0.094

*NI, novelty input; TC, trait creativity.*

**FIGURE 1:**
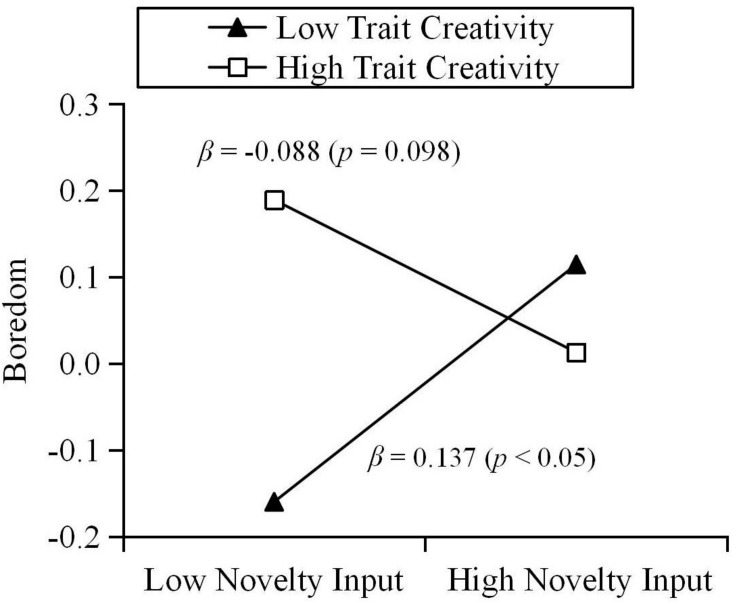
Moderation effect of trait creativity on the relationship between novelty input and boredom.

**FIGURE 2:**
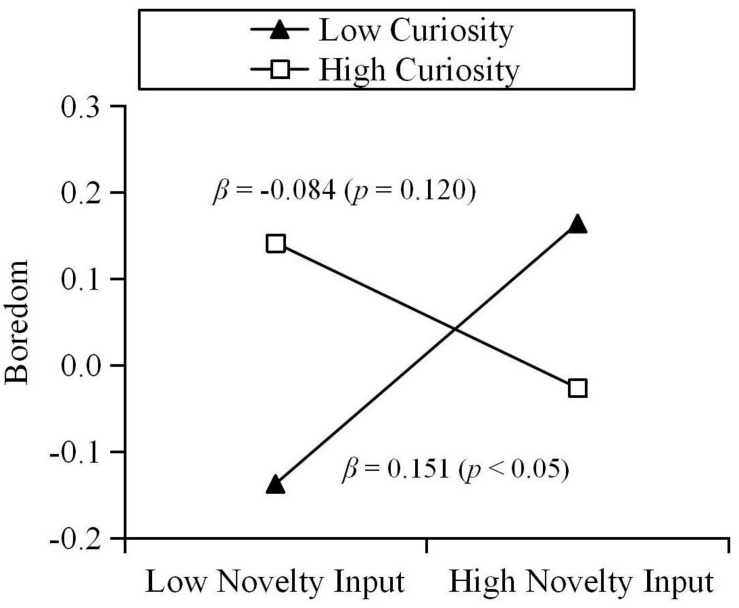
Moderation effect of curiosity on the relationship between novelty input and boredom.

**FIGURE 3:**
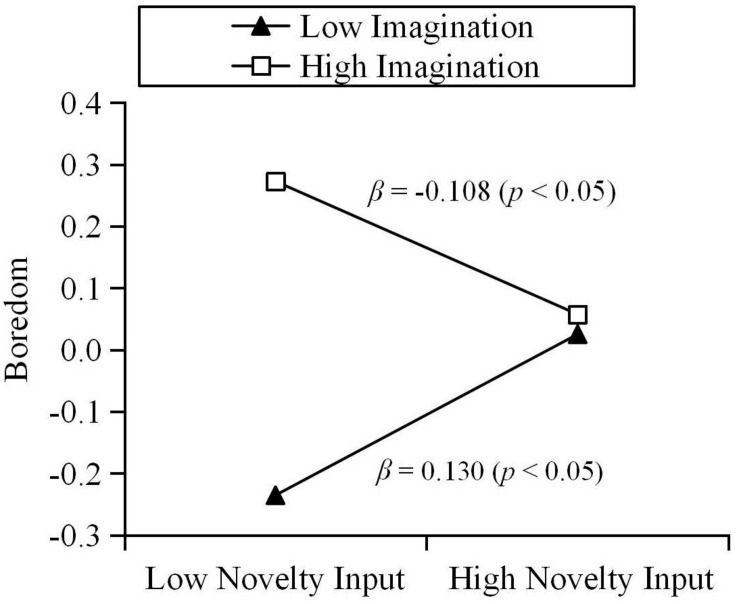
Moderation effect of imagination on the relationship between novelty input and boredom.

The results show that trait creativity moderated the relationship between novelty input and boredom during quarantine (β = −0.113, *p* < 0.01). Results of a simple slope test further revealed that, for individuals with low creativity, novelty input could positively predict state boredom. For individuals with high creativity, the relationship between novelty input and state boredom was not significant (see Figure 1). Specifically, the various components of trait creativity were used as moderators. The results are as follows: curiosity moderated the relationship between novelty input and boredom during quarantine (β = −0.117, *p* < 0.01). Results of a simple slope test further revealed that, for individuals with low curiosity, novelty input could positively predict state boredom. For individuals with high curiosity, the relationship between novelty input and state boredom was not significant (see Figure 2). In addition, imagination moderated the relationship between novelty input and boredom during quarantine (β = −0.119, *p* < 0.01). Results of a simple slope test further revealed that, for individuals with low imagination, novelty input could positively predict state boredom. For individuals with high imagination, novelty input could negatively predict state boredom (see Figure 3). Challenge and risk-taking have no significant effect on the relationship between novelty input and boredom (β = −0.069, *p* = 0.100; β = −0.066, *p* = 0.094).

### Moderation Effect of Trait Creativity on the Relationship Between Novelty Output and Boredom

The results of the moderation analysis with selected state boredom as the dependent variable, novelty output as the independent variable, and trait creativity (and its components) as a moderator are presented in Table 3.

**TABLE 3:** Results of moderation analysis with boredom as the dependent variable, novelty output as the independent variable, and trait creativity as the moderator.

Interaction effect	Coefficient	*SE*	*t*	*P*
**Trait creativity as moderating variable**				
NO × TC	−0.116	0.035	−3.306	0.001
**Indicators of trait creativity as moderating variables**				
NO × curiosity	−0.124	0.036	−3.403	0.001
NO × imagination	−0.108	0.034	−3.197	0.002
NO × challenge	−0.083	0.040	−2.085	0.038
NO × risk-taking	−0.090	0.037	−2.409	0.016

*NO, novelty output; TC, trait creativity.*

**FIGURE 4:**
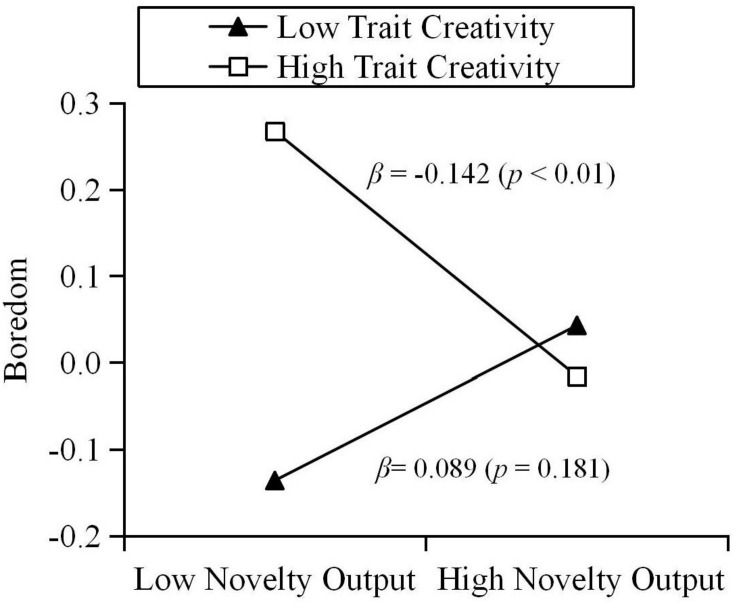
Moderation effect of trait creativity on the relationship between novelty output and boredom.

The results show that trait creativity moderated the relationship between novelty output and boredom during quarantine (β = −0.116, *p* < 0.01). Results of a simple slope test further revealed that, for individuals with high creativity, novelty output could negatively predict state boredom. For individuals with low creativity, the relationship between novelty output and state boredom was not significant (see Figure 4). Specifically, the various components of trait creativity were used as moderators, they had the same effect on the relationship between novelty output and state boredom. Curiosity moderated the relationship between novelty output and boredom during quarantine (β = −0.124, *p* < 0.01). Results of a simple slope test further revealed that, for individuals with high curiosity, novelty output could negatively predict state boredom (β_*simple*_ = −0.138, *p* < 0.01). For individuals with low curiosity, the relationship between novelty output and state boredom was not significant (β_*simple*_ = 0.109, *p* = 0.106). Imagination moderated the relationship between novelty output and boredom during quarantine (β = −0.108, *p* < 0.01). Results of a simple slope test further revealed that, for individuals with high imagination, novelty output could negatively predict state boredom (β_*simple*_ = −0.148, *p* < 0.01). For individuals with low imagination, the relationship between novelty output and state boredom was not significant (β_*simple*_ = 0.069, *p* = 0.277). Challenge moderated the relationship between novelty output and boredom during quarantine (β = −0.083, *p* < 0.05). Results of a simple slope test further revealed that, for individuals with high challenge, novelty output could negatively predict state boredom (β_*simple*_ = −0.119, *p* < 0.05). For individuals with low challenge, the relationship between novelty output and state boredom was not significant (β_*simple*_ = 0.047, *p* = 0.477). Risk-taking moderated the relationship between novelty output and boredom during quarantine (β = −0.090, *p* < 0.05). Results of a simple slope test further revealed that, for individuals with high risk-taking propensity, novelty output could negatively predict state boredom (β_*simple*_ = −0.104, *p* < 0.05). For individuals with low risk-taking propensity, the relationship between novelty output and state boredom was not significant (β_*simple*_ = 0.076, *p* = 0.272).

## Discussion

Since the quarantine environment cannot be exited or changed, individuals seek to alleviate boredom by adopting boredom coping strategies (Haager et al., 2016). When the boredom signal appears, it means that the individual needs to seek out more meaningful and satisfying alternative goal activities (Struk et al., 2016). Engaging in such activities can successfully respond to the boredom signal, thereby essentially reducing the sense of boredom (Danckert et al., 2018a; Struk et al., 2020). This study explored the relationship between novelty-seeking behavior (novelty input and novelty output) and boredom during home quarantine. In contrast to Hypothesis 1, as two behavioral coping strategies, novelty input and novelty output have no significant negative correlation with boredom during quarantine. This result is similar to that of previous research on the relationships between coping strategies and boredom (e.g., Eren, 2016). This means that as a type of boredom coping strategy, novelty seeking may not be a meaningful and satisfactory alternative goal activity for everyone. Its role in alleviating boredom may have boundary conditions.

However, we found that a link between novelty-seeking behavior and boredom is visible among individuals who exhibit specific trait creativity. Trait creativity and its four dimensions play a moderating role in the relationship between novelty output and individual state boredom. Thus, Hypothesis 3 is supported. Specifically, for individuals with high creativity, novelty output negatively predicts their boredom during quarantine. For individuals with low creativity, novelty output does not predict their boredom during quarantine. The results show that as a boredom coping strategy, novelty output is more effective for individuals with traits of high creativity. The reasons for this result may be as follows. Since meaning is an important part of the creative behavior process (Sääksjärvi and Gonçalves, 2018), individuals may gain a sense of meaning from the process of creating a novel output (Kaufman, 2018), thereby alleviating the emptiness felt in relation to doing nothing at home. However, the process of creative behavior entails the consumption of cognitive resources (Chae and Choi, 2018). Individuals with low creativity may consume more cognitive resources compared to individuals with high creativity when engaging in novelty input behavior, which may lead to a lack of cognitive resources. However, such over-challenging activities cannot alleviate the boredom (Skowronski, 2012; Vodanovich and Watt, 2016). When people engage in a creative activity they may enter a state of flow, in which they feel excited and experience optimal arousal (Csikszentmihalyi, 1990, 1996; Kaufman, 2018). Since achieving novelty output is more difficult for low creativity individuals, they are more likely feel frustrated rather than entering flow during this process (Kaufman, 2018).

Similarly, trait creativity plays a moderating role in the relationship between novelty input and individual state boredom. Thus, Hypothesis 2 is supported. However, contrary to our expectations, for individuals with low creativity, novelty input positively predicts their boredom during quarantine. For individuals with high creativity, novelty input does not predict their boredom during quarantine. This result can be explained based on the moderation effect of the trait creativity components. Due to the fact that they are lower in curiosity, the purpose of seeking novelty input for individuals with low creativity might be to cope with boredom by distracting their attention from boring situations. Thus, the passive input of novelty only improves the individual’s arousal state, without their gaining a sense of meaning. Frankl and Lasch (1992) suggested that the emptiness following feelings of meaninglessness leads to boredom. Although obtaining novel stimuli will temporarily relieve the individual’s boredom, in the long run this rapid and meaningless boredom coping strategy may aggravate their boredom (Eastwood et al., 2007; Nett et al., 2011; Elpidorou, 2017). We found that for individuals with low imagination, novelty input predicts their boredom positively, but for individuals with high imagination, novelty input predicts their boredom negatively. Imagination, which is one component of human experience, can enrich the individual’s sensory perception (Gozli, 2020). Individuals with high imagination might better perceive the non-quarantined environment in the process of novelty input, thereby relieving their monotony, and boredom. In addition, in the process of acquiring novel information, highly imaginative individuals might perform more extensive cognitive processing and integration of information (Abraham, 2016) and find solutions to certain problems or obtain creative inspiration (Scopelliti et al., 2014; Abraham, 2016). Therefore, novelty input is more active and meaningful for these individuals.

Game (2007) suggested that, compared with disengagement strategies, engagement strategies are more beneficial for individuals to relieve boredom. The results of our study also partially support this view. As a behavioral-avoidance strategy, novelty input only takes the form of changing attention and raising the level of arousal to cope with boredom. It does not change the current boredom situation. Thus, if we passively receive stimulation without being aware of our desires and interacting with the current environment, this novelty-seeking behavior will be counterproductive (Eastwood et al., 2007). Especially in the quarantine environment, individuals may experience depression and anxiety (Tang et al., 2020). In this situation, if people cannot cope with boredom well, and use mobile phones to obtain information more frequently to alleviate boredom, it may lead to problematic smartphone use (Elhai et al., 2017). When individuals engage in creative behaviors their boredom related to being in quarantine might change. Furthermore, individuals with high creativity have a greater sense of challenge and adventure, so may have a greater desire to engage in novel behaviors during quarantine and obtain satisfaction from this behavior. Therefore, compared with novelty input, novelty output may alleviate the quarantine boredom experienced by highly creative individuals. The present study shows that when dealing with boredom it is vital to be aware of your inner desires and use active and creative coping strategies to replace passive entertainment (Eastwood et al., 2007; Zhou et al., 2012). For individuals with low creativity, novelty seeking seems not to be a good way to relieve boredom during home quarantine. However, this result does not mean that low creativity individuals cannot alleviate boredom, because there are still some ways to cope with boredom that this study has not explored. For example, Nett et al. (2010) found that cognitive strategies (e.g., positive reappraisal) may be the most beneficial for students to reduce academic boredom.

This study is subject to several limitations, which also offer avenues for future research. First, novelty-seeking behavior may cause the individual’s state boredom during quarantine to fluctuate across short spans of time. Collecting data on novelty-seeking behavior and state boredom in a cross-sectional manner is likely to obscure these temporal variations (Skowronski, 2012). Second, although we establish a moderation model based on theory and previous research, our non-experimental and cross-sectional design prevents us from making causal inferences. Therefore, we were unable to ascertain the causal relationships between novelty-seeking behavior and state boredom. The findings obtained in this research should thus be interpreted with caution. Future research can use empirical sampling to address this limitation. For example, researchers can ask quarantined participants to report their boredom levels and novelty-seeking behavior at random points over time using their mobile phone (Nett et al., 2011). If novelty-seeking behaviors immediately precede boredom fluctuations, this provides a solid basis for inferring causality. Moreover, such an approach would avoid methodological problems such as the common-method bias of having individuals estimate their overall level of quarantine boredom in a one-time questionnaire (Skowronski, 2012). Third, it takes a certain amount of energy and willpower to deal with boredom through novelty output, but this study did not measure variables that may affect these factors. Fourth, the sample source of this study is relatively single, and the participants are all college students. Previous studies have found that boredom proneness (trait boredom) is negatively correlated with individual age (Isacescu et al., 2017; Boylan et al., 2020). Therefore, compared with older people, this group may have higher boredom proneness and more likely to be bored in quarantine environments. Future studies should test the generalizability of the research findings with different samples from other groups or geographic regions. Fifth, we did not collect our data during the peak of the COVID-19 pandemic in China. Our participants completed the questionnaire over 2 months after the strict close-off management relaxed. They were asked to recall their situation during strict closed-off community management in answering the questions. However, the management was in effect during this period, and universities still had not opened; thus, the college students we selected were still in a quarantine situation when they answered the questions. In addition, boredom is widespread among college students (Pekrun et al., 2010), and future research can focus on exploring the differences in boredom coping strategies of students of different majors.

## Conclusion

Our findings indicate that trait creativity is an important factor affecting the relationship between individual novelty-seeking behavior and boredom. Novelty input and novelty output have different effects on the boredom of individuals with different levels of creativity during home quarantine. For individuals with high creativity, there is a negative relationship between novelty output and state boredom; for individuals with low creativity, especially individuals with low curiosity and low imagination, novelty input may have a negative impact on them. However, as a way of coping with boredom, novelty input is not completely useless. For individuals with high imagination, there is a positive relationship between novelty output and state boredom. The results of this study suggest that, due to variations in the attributes of distinct boredom coping strategies, novelty-seeking behavior may have different effects on individuals with different traits. Individuals should avoid excessively engaging in novelty input behaviors in order to escape boring situations.

## Data Availability Statement

The raw data supporting the conclusions of this article will be made available by the authors, without undue reservation.

## Ethics Statement

The studies involving human participants were reviewed and approved by the Ethics Institutional Review Board of Central China Normal University. The patients/participants provided their written informed consent to participate in this study.

## Author Contributions

ZL, QZ, ZZ, QY, SL, and SC designed the study and interpreted the results. ZL collected the data for the study and wrote the first draft of the manuscript. ZL, QZ, ZZ, and QY edited the first draft of the manuscript. All authors approved the final version of the manuscript.

## Conflict of Interest

The authors declare that the research was conducted in the absence of any commercial or financial relationships that could be construed as a potential conflict of interest.
